# Effect of 2-Chloro-Substitution of Adenine Moiety in Mixed-Ligand Gold(I) Triphenylphosphine Complexes on Anti-Inflammatory Activity: The Discrepancy between the *In Vivo* and *In Vitro* Models

**DOI:** 10.1371/journal.pone.0082441

**Published:** 2013-11-27

**Authors:** Jan Hošek, Ján Vančo, Pavel Štarha, Lenka Paráková, Zdeněk Trávníček

**Affiliations:** 1 Department of Inorganic Chemistry, Regional Centre of Advanced Technologies and Materials, Faculty of Science, Palacký University, Olomouc, Czech Republic; 2 Department of Human Pharmacology and Toxicology, Faculty of Pharmacy, University of Veterinary and Pharmaceutical Sciences, Brno, Brno, Czech Republic; Martin-Luther-Universität Halle-Wittenberg, Germany

## Abstract

A series of gold(I) triphenylphosphine (PPh_3_) complexes (1–9) involving 2-chloro-*N*6-(substituted-benzyl)adenine derivatives as N-donor ligands was synthesized and thoroughly characterized by relevant methods, including electrospray-ionization (ESI) mass spectrometry and multinuclear NMR spectroscopy. The anti-inflammatory and antiedematous effects of three representatives **1**, 5 and 9 were evaluated by means of *in vitro* model based on the expression of pro- and anti-inflammatory cytokines and influence of the complexes on selected forms of matrix metalloproteinases secreted by LPS-activated THP-1 monocytes and *in vivo* model evaluating the antiedematous effect of the complexes in the carrageenan-induced rat hind-paw edema model. In addition to the pharmacological observations, the affected hind paws were *post mortem* subjected to histological and immunohistochemical evaluations. The results of both *in vivo* and *ex vivo* methods revealed low antiedematous and anti-inflammatory effects of the complexes, even though the *in vitro* model identified them as promising anti-inflammatory acting compounds. The reason for this discrepancy lies probably in low stability of the studied complexes in biological environment, as demonstrated by the solution interaction studies with sulfur-containing biomolecules (cysteine and reduced glutathione) using the ESI mass spectrometry.

## Introduction

The mixed-ligand gold(I) complexes, involving the derivatives of phosphine, are in the scope of chemists for several reasons. One of these includes the ability of chiral gold(I)-phosphine complexes to involve in the catalytic asymmetric gold-catalyzed reactions providing versatile routes to enantiomerically enriched carbo- and hetero-cycles [1–3]. The other reasons embody the ability of gold(I)-phosphine complexes to interact with biological systems and act as biologically active agents, dominantly showing the cytotoxic [4–6], biocidal [7], or anti-inflammatory activities [8–10]. Over the years, since the oligodynamic effect of gold and its compounds was described by von Nägeli et al. [11], many diverse mechanisms standing behind the biological activities of gold(I) complexes were discovered. 

It has been shown that the gold(I) complexes, exhibiting the cytotoxic and antitumor activities, do not primarily target DNA [12] (as compared to platinum antitumor metallodrug cisplatin), but their main targets are the components of proteasome [13]. In addition, it was shown that the gold(I) species are able to take part in the redox cycling and interact with cellular redox processes by targeting mitochondria [14–16], leading to the decrease of the ATP concentration by uncoupling of oxidative phosphorylation, and thus inhibition of the oxidative ADP phosphorylation [17,18]. However, probably the major impact of gold(I) complexes on redox homeostasis of cancer cells is the inhibition of the cytosolic and mitochondrial thioredoxin reductase (TrxR) system [19–21]. The ability of gold(I) species to interact with the active site of thioredoxin reductase can be clarified sufficiently by the application of the Pearson’s principle of hard and soft acids and bases, while the gold(I) species as a soft acid tend to bind with the soft base ligands [19]. Therefore it prefers the binding to selenolate groups of TrxR, subsequently leading to the inhibition of its activity both in cytosol and mitochondria, leaving the similar system of glutahione reductase, containing the thiolate groups in the active site, unaffected [19,22] up to high concentrations. The sum of all the above mentioned effects, the TrxR inhibition, disturbance of mitochondrial respiration, increased production of reactive oxygen species by redox cycling, mitochondrial swelling, and decreasing in the mitochondrial membrane potential, subsequently lead to apoptosis [4]. Additionally, it has been also found that Auranofin inhibits TrxR in a p53-independent manner [23]. 

In addition to the alterations of the GSH and TrxR systems, the anti-inflammatory active compounds like Auranofin showed the ability to induce the HO-1 expression by activating Keap1/Nrf2 signaling via Rac1/iNOS induction and MAPK activation [24]. It has been also shown that Auranofin can inhibit the activation of STAT3, NF-κB, and the homodimerization of toll-like receptor 4 [25–27]. 

The biological perspective of gold(I) complexes as anti-inflammatory and antiedematous agents [8–10], represented by Auranofin® clinically utilized as a drug for the treatment of rheumatoid arthritis [28], led us previously to prepare a series of gold(I) triphenylphosphine complexes involving various *N*6-(substituted-benzyl)adenine derivatives as adenine-based *N*-donor ligands [10]. It has been shown that these compounds strongly reduced the production of pro-inflammatory cytokines TNF-α, IL-1β and HMGB1 without the influence on the secretion of anti-inflammatory cytokine IL-1RA in the LPS-activated macrophages. Several representatives of this group also reduced the formation of edema caused by the application of polysaccharide λ-carrageenan *in vivo*. All the previously studied gold(I) complexes exhibit similar or better effects as compared to Auranofin in equitoxic doses but lower cytotoxicity than Auranofin.

It is generally known, that the substitution of organic ligands, such as the above mentioned *N*6-benzyladenines, involved in structures of transition metal complexes provide different rate or even type of biological activity of such complexes [29,30]. This fact motivated us to prepare a series of structurally analogical gold(I) complexes of the [Au(L_n_)(PPh_3_)] type with a different type of the *N*6-benzyladenine derivatives. Concretely, we used 2-chloro-*N*6-(substituted-benzyl)adenine derivatives (HL_n_), which differ in the substitution at the C2 atom as compared to those involved in our previous work dealing with gold(I) triphenylphosphine complexes involving *N*6-(substituted-benzyl)adenine-based.

## Materials and Methods

This study was carried out in strict accordance with the recommendations in the Guide for the Care and Use of Laboratory Animals of the National Institutes of Health. The protocol was approved by the Expert committee on the protection of animals against cruelty at the University of Veterinary and Pharmaceutical Sciences (Permit Number: 113/2010, authorization for the use of animals No. 407/2011-30). To minimize the suffering of laboratory animals, all pharmacological interventions were done under anaesthesia. The animal tissues for *ex vivo* experiments were taken post mortem, immediately after all animals were sacrificed by cervical dislocation.

### Chemicals and Biochemicals

H[AuCl_4_]·3H_2_O (Acros Organics, Pardubice, Czech Republic), triphenylphosphine (PPh_3_; (Sigma-Aldrich Co., Prague, Czech Republic), NaOH (Sigma-Aldrich Co., Prague, Czech Republic) and all solvents (acetone, benzene, diethyl ether, dimethyl sulfoxide, N,N'-dimethylformamide, hexane, water; Fisher-Scientific, Pardubice, Czech Republic) were obtained from the mentioned commercial sources and were used without further purification. The 2-chloro-*N*6-benzyladenine derivatives HL_1_-HL_9_ were synthesized according to the previously published procedure [31] and characterized by elemental analysis, FT-IR, Raman and ^1^H and ^13^C NMR spectroscopy to prove their composition and purity. The [AuCl(PPh_3_)] complex was prepared as described previously [32,33]. 

RPMI 1640 medium and penicillin-streptomycin mixture were purchased from Lonza (Verviers, Belgium). Phosphate-buffered saline (PBS), fetal bovine serum (FBS), phorbol myristate acetate (PMA), Auranofin (98%≤), erythrosin B, and *Escherichia coli* 0111:B4 lipopolysaccharide (LPS) were purchased from Sigma-Aldrich (Steinheim, Germany). Cell Proliferation Reagent WST-1 was obtained from Roche (Mannheim, Germany). Instant ELISA Kits (eBioscience, Vienna, Austria) were used to evaluate the production of TNFα and IL-1β.

### Chemistry

The acetone solutions of [AuCl(PPh_3_)] (1 mmol in 10 mL) and the appropriate *N*6-(benzyl)adenine derivative (HL_1_-HL_9_; 1 mmol in 50 mL) were mixed together and consecutively, 1 mL of 1M NaOH was poured into the reaction mixture. The mixture was stirred for 4 h, after that NaCl was filtered off. The colourless filtrate was evaporated to dryness and the residue was dissolved in benzene (10 mL). The solution was added drop wise into hexane (200 mL). The white solid, which formed, was filtered off, washed with acetone (5 mL) and diethyl ether (10 mL) and dried at 40 °C under an infrared lamp. 

The results of elemental analysis, electrospray-ionization (ESI) mass spectrometry, ^1^H, ^13^C and ^31^P NMR, FT-IR and Raman spectroscopies, as well as thermogravimetric (TG/DTA) and molar conductivity measurements clearly confirmed the purity and composition of the obtained gold(I) complexes **1**–**9** (see Figures S1 and S2).

### Physical Measurements

Elemental analyses (C, H, N) were performed on a Flash 2000 CHNO-S Analyser (Thermo Scientific). The chlorine contents were determined using the Schöniger method [34]. Conductivity measurements of 10^-3^ M N,N'-dimethylformamide (DMF) and 10^-3^ M methanol solutions were carried out using a conductometer 340i/SET (WTW) at 25 °C. FT-IR spectra were recorded on a Nexus 670 FT-IR spectrometer (ThermoNicolet) using ATR technique at the 400–4000 cm^-1^ and 150–600 cm^-1^ regions. A Raman spectrometer Nicolet NXR 9650 equipped with the liquid nitrogen cooled NXE Genie germanium detector (ThermoNicolet) was used to record Raman spectra in the region of 150–3750 cm^-1^. ^1^H, ^13^C and ^31^P NMR spectra of DMF-*d*_*7*_ solutions were measured on a Varian 400 MHz NMR spectrometer at 300 K with the tetramethylsilane (SiMe_4_) used as an internal reference standard (for ^1^H and ^13^C spectra) and 85% H_3_PO_4_ (for ^31^P). Mass spectra of the methanol solutions were obtained by an LCQ Fleet ion trap mass spectrometer by the positive mode electrospray ionization (ESI+) technique (Thermo Scientific). The theoretic values were calculated by the QualBrowser software (version 2.0.7, Thermo Fisher Scientific). Thermogravimetric (TG) and differential thermal (DTA) analyses were performed using a thermal analyzer Exstar TG/DTA 6200 (Seiko Instruments Inc.) in dynamic air conditions (100 mL min^-1^) between room temperature (*ca* 25 °C) and 900 °C (gradient 2.5 °C min^-1^). 

### Maintenance and Preparation of Macrophages

For the determination of biological activities *in vitro* (cytotoxicity testing, induction of inflammatory response and the evaluation of cytokine secretion), we used the human monocytic leukemia cell line THP-1 (ECACC, Salisbury, UK). The cells were cultivated at 37 °C in RPMI 1640 medium supplemented with 2 mM of l-glutamine, 10% FBS, 100 U/mL of penicillin and 100 µg/mL of streptomycin in a humidified atmosphere containing 5% CO_2_. Stabilized cells (3^rd^–15^th^ passage) were split into microtitration plates to get a concentration of 100.000 cells/mL and the differentiation to macrophages was induced by 50 ng/mL PMA dissolved in DMSO, as described previously [35].

### Cytotoxicity Testing

THP-1 cells (floating monocytes, 500.000 cells/mL) were incubated in 100 μL of serum-free RPMI 1640 medium and seeded into 96-well plates in triplicate at 37 °C. The measurements were done 24 h after the treatment with 0.039, 0.156, 0.625, 2.5, and 10 μM solutions of the tested compounds **1**, **5** and **9** dissolved in dimethyl sulfoxide (DMSO). Viability was determined by the WST-1 test according to the manufacturer’s manual. The amounts of created formazan (which correlate to the number of metabolically active cells in the culture) were calculated as a percentage of control cells, which were treated only with DMSO (100% viability). The cytotoxic LD_50_ concentrations of the tested compounds were determined by the data from the equation generated by the KURV+ Version 4.4b software (Conrad Button Software, Arlington, WA, USA).

### Drug Treatment and Induction of Inflammatory Response

Differentiated macrophages were pretreated with 100 nM or 600 nM solution of **1**, 5 and 9 in 0.1% DMSO, 100 nM solution of Auranofin in 0.1% DMSO, and with 0.1% DMSO solution itself (*vehicle*) for 1 h; the given concentrations of the tested compounds lack cytotoxic effect (cell viability >94%). The inflammatory response was triggered by adding of 1.0 µg/mL LPS dissolved in water to pre-treated macrophages. Control cells were pre-treated by DMSO only and subsequently incubated without the addition of LPS, thus serving as a source of the basal expression of pro-inflammatory cytokines. Each experiment was repeated three times.

### Evaluation of Cytokine Secretion by ELISA

Macrophages, which were pretreated with the tested compounds for 1 h, were incubated with LPS for next 24 h. After this period, medium was collected and the concentration of TNFα, and IL-1β was measured by Instant ELISA kit according to manufactures’ manual.

### Zymography

Conditioned media obtained by the same way as for cytokines evaluation were used for the measurement of matrix-metalloproteinases (MMP) activity by zymography, as described previously [36]. Briefly, 20 μL of collected medium was loaded into a non-denaturating 12% polyacrylamide gel impregnated by 0.1% (w/v) gelatin. After the electrophoresis experiments, sodium dodecyl sulfate (SDS) from gels was washed out by 2.5% Triton X100 and the gels were incubated in developing buffer (50 mM Tris pH 8.8, 5 mM CaCl_2_, 3 mM NaN_3_ and 0.5% Triton X100) at room temperature (*ca* 23 °C) for 30 min and at 37 °C overnight (16–20 h). Gels were stained by Coomassie blue. Intensities of digested regions were calculated densitometrically using the AlphaEasy FC 4.0.0 software (Alpha Innotech, USA).

### Animals

Wistar - SPF (6–8 weeks male) rats were obtained from the AnLab, Ltd., Prague. The animals were kept in plexiglass cages at the constant temperature of 22±1 °C, and relative humidity of 55±5% for at least 1 week before the experiment. They were given food and water *ad libitum*. All experimental procedures were performed according to the National Institutes of Health (NIH) Guide for the Care and Use of Laboratory Animals. In addition, all tests were conducted under the guidelines of the International Association for the Study of Pain [37]. After the one week adaptation period, male Wistar-SPF rats (200–250 g) were randomly assigned to the groups (n = 10). The control group received 25% DMSO (v/v in water for injections PhEur, intraperitoneal; i.p.). The other three groups include a carrageenan-treated, an Auranofin positive control (Auranofin + carrageenan) and indomethacin positive control (indomethacin + carrageenan) groups [10].

### Carrageenan-induced Hind Paw Edema and Ex vivo Histological and Immunohistochemical Evaluation

The carrageenan-induced hind paw edema model was used for the determination of the anti-inflammatory activity [10]. Animals were *i.p.* pretreated with the complexes **1**, **5** and **9** (10 mg/kg), Auranofin (10 mg/kg; positive control), indomethacin (5 mg/kg; positive control) or 25% DMSO (v/v in water for injections PhEur), 30 min prior to the injection of 1% λ-carrageenan (50 μL) into the plantar side of right hind paws of the rats. The paw volume was measured immediately after the carrageenan injection and during the next 6 h after the administration of the edematogenic agent using a plethysmometer (model 7159, Ugo Basile, Varese, Italy). The degree of swelling induced was evaluated by the percentage of change of the volume of the right hind paw after the carrageenan treatment from the volume of the right hind paw before carrageenan treatment. The data were combined afterwards for all 10 animals within the each experimental group and subjected to statistical evaluation by the one-way ANOVA with *post-hoc* Tukey test of significance. 

All animals were sacrificed by cervical dislocation, and immediately after that, the affected hind paws were separated and underwent the process of dehydration and fixation, and were embedded into paraffin blocks by means of standard protocols [38]. The histopathological changes, like infiltration of different skin elements and deeper laying tissues by the white blood cells (dominantly neutrophils and lymphocytes) stained by the standard H&E-staining and Gömöri trichrome staining, were evaluated. In addition to the classical histological investigation, the immunohistochemical detection of apoptosis (caspase 3 and TUNEL), TNF-α, interleukin 6 (IL-6) and selectin E (CD62E) was performed using the Abcam® rat monoclonal abtibodies.

### Interactions with Cysteine and Reduced Glutathione Assessed by ESI Mass Spectrometry

The interaction experiments between the selected representative complex **5** and the mixture of physiological levels of cysteine and glutathione were performed on a ThermoFinnigan LTQ Fleet Ion-Trap mass spectrometer, using the positive ionization mode. The FIA method was used to introduce the reaction system (100 μL spikes) into the mass spectrometer, while the mixture of 10 mM ammonium acetate solution and methanol (10:90 v/v) was used as a mobile phase. The ESI- source was set up as follows: source voltage was 4.48 kV, the vaporizer temperature was 160°C, the capillary temperature was 275 °C, the sheath gas flow rate was 20 L/min, and auxiliary gas flow rate was 5 L/min. The system was calibrated according to the manufacturer specifications and no further tuning was needed.

## Results and Discussion

### Characterization of the Complexes

**Figure 1:**
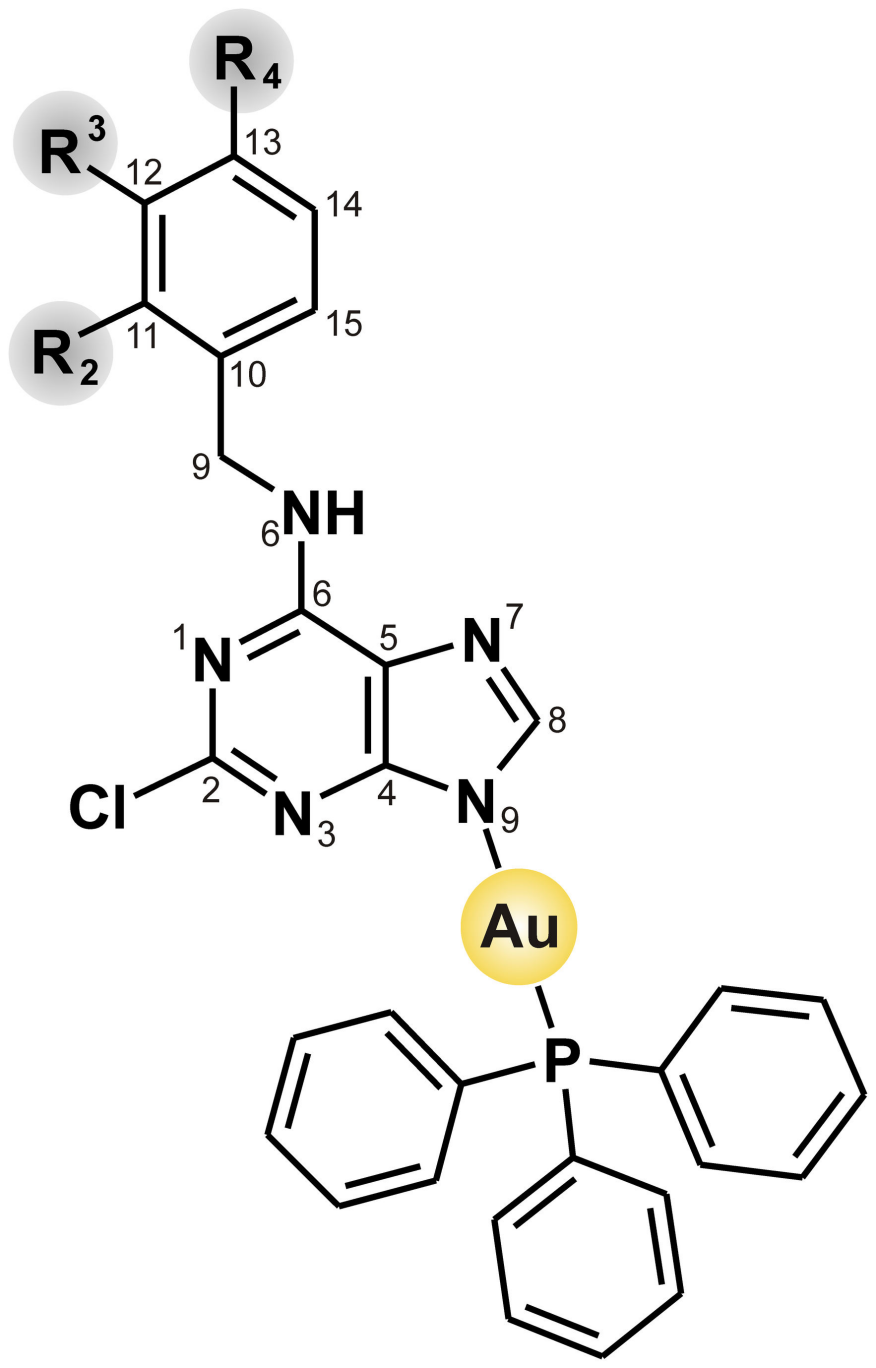
Schematic representation of complexes 1–9. R symbolizes hydrogen for HL_1_ and 1, 3-fluoro for HL_2_ and 2, 2-chloro for HL_3_ and 3, 3-chloro for HL_4_ and 4, 2-methoxy for HL_5_ and 5, 3-methoxy for HL_6_ and 6, 4-methoxy for HL_7_ and 7, 4-hydroxy for HL_8_ and 8 and 4-methyl for HL_9_ and 9.

The composition of the prepared complexes **1**–**9** (Figure 1) correlates with the determined content of C, H, N and Cl, since the differences between the calculated and found values were up to 0.44%. The complexes **1**−**9** are very well soluble in DMF, methanol and ethanol and partially soluble in water at room temperature. The non-electrolytic character of the complexes was proved by the molar conductivity measurements, because the obtained values of the 10^–3^ M methanolic and DMF solutions (See Figures S1 and S2) fell to the intervals characteristic for nonelectrolytes [39]. ESI+ mass spectra of 1–9 contain the [Au(HL_n_)(PPh_3_)]^+^ molecular peak, as well as that of [HL_n_+H]^+^ belonging to the free adenine-based ligand. The [Au(L_n_)(PPh_3_)+Na]^+^ adducts were detected in the spectra of **2**, **5**, **7** and **9**. The simultaneous TG/DTA analysis proved the studied compounds as non-solvated ones (thermally stable up to 135 °C), except for **6** and **7** (monohydrates, see Figure S1). 

The bands of very strong intensity, whose maxima were detected in the 1606–1613 cm^−1^ (FT-IR) and 1604–1618 cm^-1^ (Raman) regions, may be assigned to the ν(C=N)_ar_ vibration of the purine ring [40,41]. The bands found in both the FT-IR and Raman spectra between 2900 and 3000 cm^-1^, and at about 3060 cm^-1^ can be assigned to ν(C–H)_aliph_, including ν(C–H)_met_ of 5–7 and **9**, and ν(C–H)_ar_, respectively. The characteristic vibrations connected with the phenyl ring substitution, in particular ν(C_ar_-F) for **2** at 1264 cm^-1^, and ν(C_ar_-Cl) for 3 and 4 at *ca* 1160 cm^-1^ , were detected as well. The strong bands observed in the 996–1003 cm^-1^ (FT-IR) and 1000–1003 cm^-1^ (Raman) regions may be ascribed to the C2-Cl vibrations observed in the spectra of all the complexes. The maxima at 539–545 cm^-1^ (FT-IR) and 531–555 cm^-1^ (Raman) may be assigned to the ν(Au–N) [42,43] stretching vibrations, while the maxima at 342–365 cm^-1^ (FT-IR) and 345–372 cm^-1^ (Raman) belong to the ν(Au–P) [44,45] vibrations of 1–9. The complete results of the FT-IR and Raman spectroscopies are given in Figures S1 and S2.

The obtained NMR spectroscopy results gave evidence of the composition of the gold(I) complexes by means of the presence of both types of ligands in the structure of 1–9 as well as their coordination mode. The highest ^13^C NMR coordination shifts (*Δ*δ = *δ*_*complex*_^_^ *δ*_*ligand*_, ppm; Table 1) were determined for the C4 and C8 atoms shifted by 1.73–5.73 ppm upfield, and 7.14–9.06 ppm downfield, respectively (see Table 1). This is caused by the fact that these atoms are adjacent to the N9 coordination site. The signals observed in the ^1^H NMR spectra for C8-H were shifted by 0.23-0.30 ppm upfield. The N6–H signals were detected as slightly shifted in the case of 1–9, while the N9–H signals were not found for all the studied complexes as expected for the deprotonated 2-chloro-*N*6-benzyladenine-based ligands symbolized as L_n_ (see Table 1). The ^31^P NMR spectra of the complexes showed singlets at 30.97–32.44 ppm (see Figure S2) as compared to the signal of free PPh_3_ (-5.96 ppm). The obtained results indicated that the gold(I) atom is coordinated by one adenine derivative coordinated through its N9 atom and one PPh_3_ molecule coordinated through phosphorus atom. 

**Table 1:** Selected ^1^H and ^13^C coordination shifts (calculated as *Δ*δ = *δ*_*complex*_ – *δ*_*ligand*_) of the prepared gold(I) complexes 1–9.

**Complex**	^1^H NMR			^13^C NMR					^31^P NMR
	N6H	C8H	N9H	C2	C4	C5	C6	C8	P
**1**	-0.51	-0.23	nd	-1.82	-1.97	0.14	-0.40	7.14	38.40
**2**	-0.36	-0.23	nd	-1.89	-1.83	0.21	-0.36	7.80	no**^*a*^**
**3**	-0.56	-0.29	nd	-1.92	-1.81	0.44	-0.27	8.97	31.41
**4**	-0.56	-0.29	nd	-1.90	-1.73	0.65	-0.12	8.95	31.37
**5**	-0.30	-0.24	nd	-1.65	-5.73	0.40	-0.10	8.81	37.81
**6**	-0.58	-0.28	nd	-2.06	-2.98	0.80	-0.07	8.38	36.93
**7**	-0.55	-0.24	nd	-1.98	-1.90	0.45	-0.19	8.48	no**^*a*^**
**8**	-0.37	-0.30	nd	-2.15	-2.29	2.00	0.39	8.65	37.34
**9**	-0.54	-0.25	nd	-2.00	-1.91	0.60	-0.07	9.06	37.48

nd = not detected; no**^*a*^** = signal not observed even after 14 hrs of the experiment due to limited solubility of the complexes in DMF-*d*_*7*_.

### Cytotoxicity to THP-1 Cells

**Figure 2:**
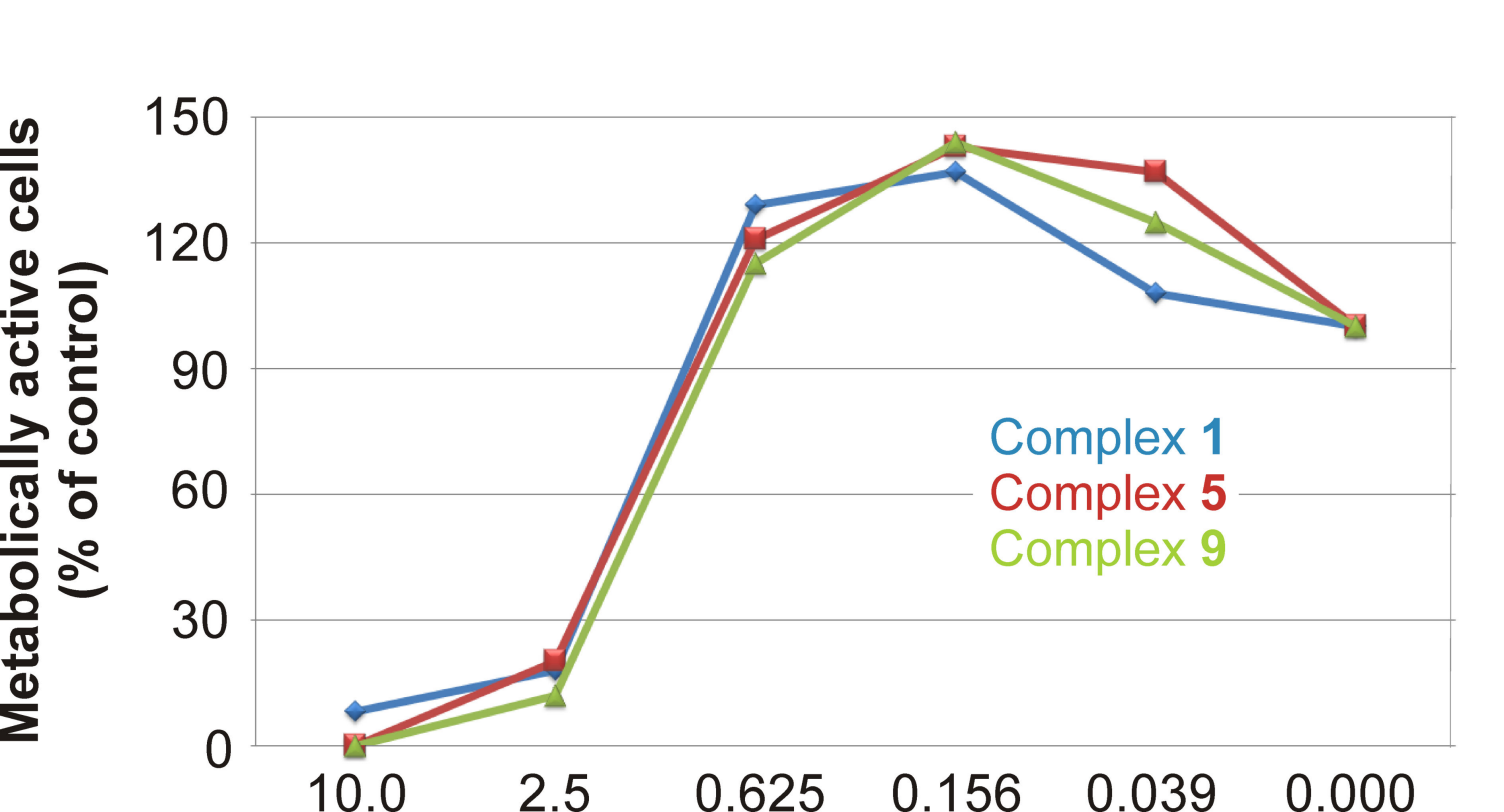
Cytotoxicity of complexes 1, 5, and 9 on THP-1 cells. THP-1 cells were treated with the decreasing concentration (10–0.039 μM) of 1, 5, and 9, respectively. The number of metabolically active cells was determined by the WST-1 test after 24 h of incubation. The viability was calculated in comparison to the vehicle-treated cells. The results are expressed as means ± S.E. for three independent experiments.

The cytotoxic effect of **1**, 5 and 9 was evaluated on the THP-1 cell line. The complexes **1**, **5** and **9** exhibit similar LD_50_ values ranged from 1.45 to 1.58 µM (Figure 2). The tested compounds demonstrated hormesis (in our case higher metabolic activity in the comparison with untreated cells) in concentrations lower than 1 µM. Similar effect was observed for other gold(I) complexes containing different *N*6-benzyladenine derivatives and triphenylphosphine moiety described previously [10]. It could be caused by soft oxidative stress induced by Au(I) atom, which results in activation of repairing and/or pro-survival processes [46].

### Cytokine Expression

**Figure 3:**
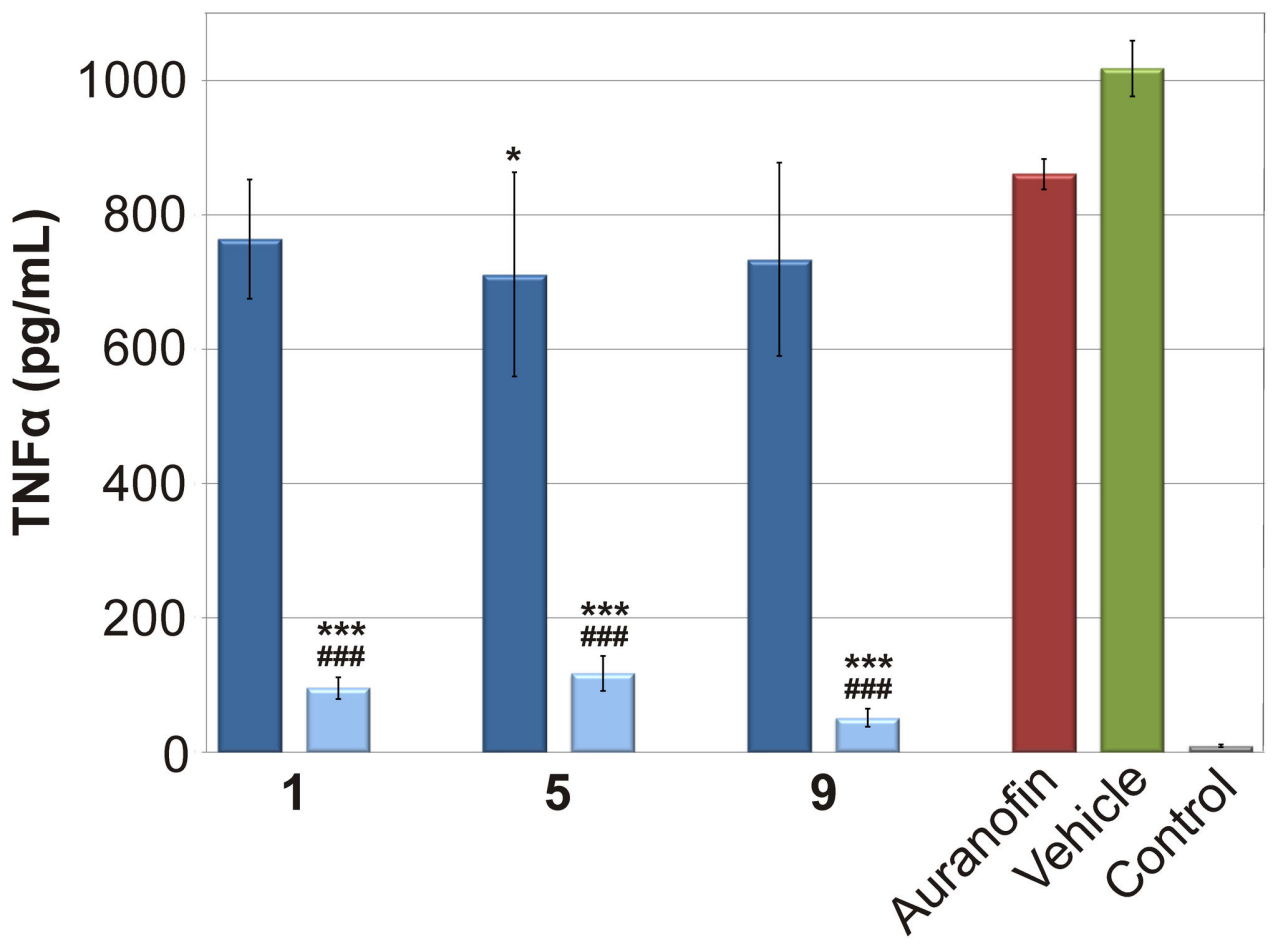
Effects of the gold(I) complexes and the reference drug Auranofin on the LPS-induced TNF-α secretion. The cells were pretreated with complexes 1, 5 and 9 (100 nM - depicted in dark blue; 600 nM - depicted in light blue), and Auranofin (100 nM), or the vehicle (DMSO) only. After 1 h of the incubation, the inflammatory response was induced by LPS [except for the control cells]. The secretion was measured 24 h after the LPS addition. The results are expressed as means for three independent experiments. * Significant difference in comparison to vehicle-treated cells (p < 0.05), *** significant difference in comparison to vehicle-treated cells (p < 0.001), ### significant difference in comparison to Auranofin-treated cells (p < 0.001).

To evaluate the anti-inflammatory potential of the tested gold(I) complexes, the expression of two typical pro-inflammatory cytokines TNF-α and IL-1β was determined *in vitro*. To better understand the activity of **1**, 5 and 9 we tested their influence on the selected pro- and anti-inflammatory cytokines in both the equitoxic (600 nM) and equimolar (100 nM) concentrations as compared to Auranofin. Our previous results indicated that gold(I) complexes with *N*6-benzyladenine derivatives as N-donor ligands were able to attenuate the LPS-induced secretion of these cytokines [10]. A gentle diminution of the TNF-α secretion (by the factor of 1.18–1.43) was observed after the pretreatment of the cells by 100 nM solutions of **1**, **5**, **9** or Auranofin (Figure 3). On the other hand, when the higher concentration of the studied complexes was used (600 nM), the production of TNF-α decreased significantly by the factor of 10.69 (for **1**), 8.71 (for **5**) and 20.07 (for **9**).

**Figure 4:**
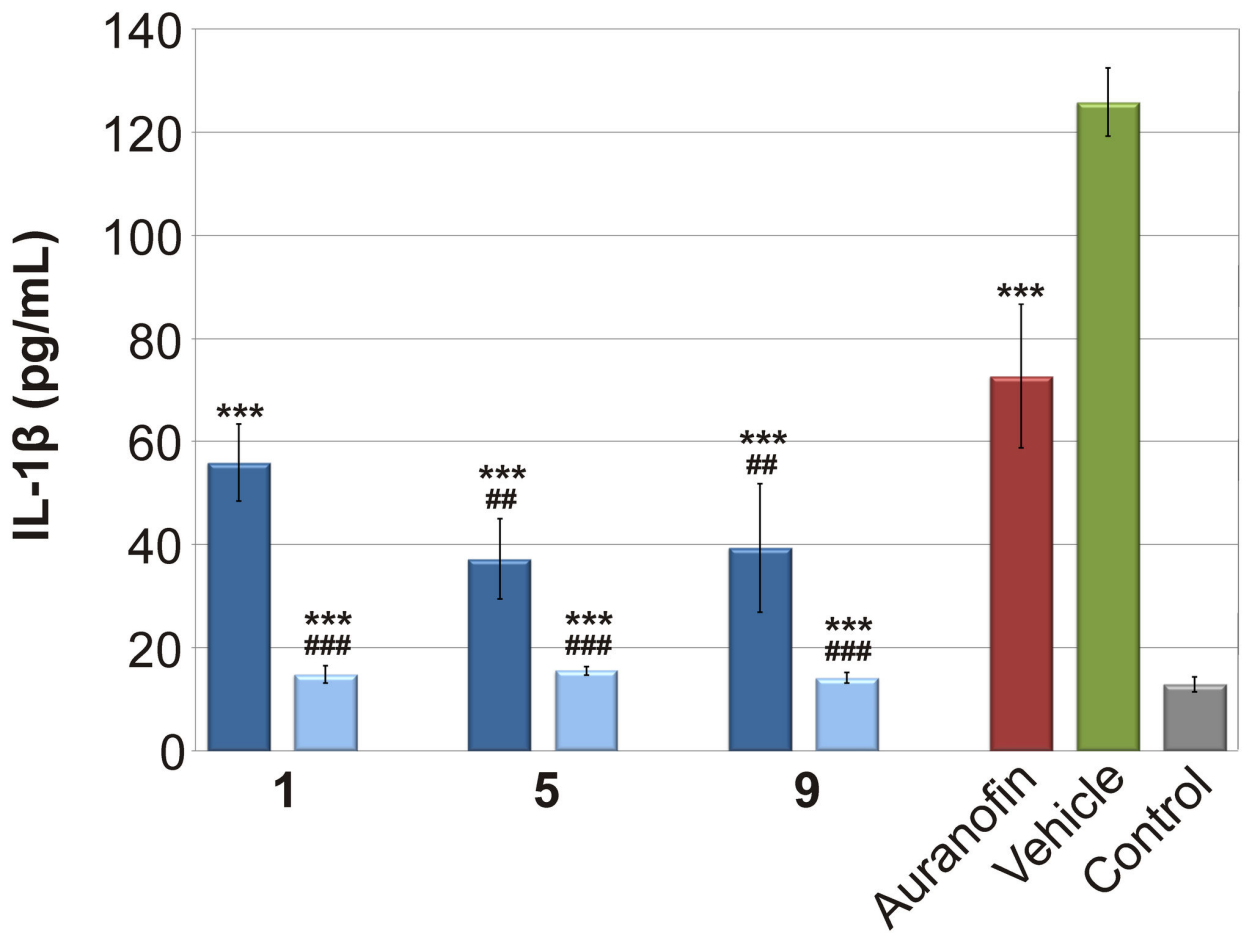
Effects of the gold(I) complexes and the reference drug Auranofin on the LPS-induced IL-1β secretion. The cells were pre-treated with complexes 1, 5 and 9 (100 nM - depicted in dark blue; 600 nM - depicted in light blue) and Auranofin (100 nM), or the vehicle (DMSO) only. After 1 h of the incubation, the inflammatory response was induced by the LPS [except for the control cells]. The secretion was measured 24 h after the LPS addition. The results are expressed as means for three independent experiments. *** Significant difference in comparison to vehicle-treated cells (*p* < 0.001), # significant difference in comparison to Auranofin-treated cells (*p* < 0.05), ## significant difference in comparison to Auranofin-treated cells (*p* < 0.01), ### significant difference in comparison to Auranofin-treated cells (*p* < 0.001).

It has been previously described that gold sodium thiomalate, i.e. sodium ((2-carboxy-1-carboxylatoethyl)thiolato)gold(I), GST, preferentially inhibits the IL-1β production rather than TNF-α [47]. This effect of gold-containing compounds was proven by this experiment, where Auranofin 1.73-times inhibited the IL-1β secretion (Figure 4). In this parameter, the tested compounds **1**, **5** and **9** were significantly more active than Auranofin and reduced the production of this cytokine by the factor of 2.25–3.38. When the higher concentration was used, the reduction reached the value of 8.12-times for **5**. These results corresponded well with our previous findings on the similar gold(I) complexes with *N*6-benzyladenine derivatives, where we noted only moderated effect of gold(I) complexes on the TNF-α secretion, but the production of IL-1β was strongly affected [10]. Newly synthesized complexes can be arranged according to their ability to reduce the production of pro-inflammatory cytokines TNF-α and IL-1β as follows: Auranofin (the least potent) < 5 < 1 < 9 (the most potent). 

**Figure 5:**
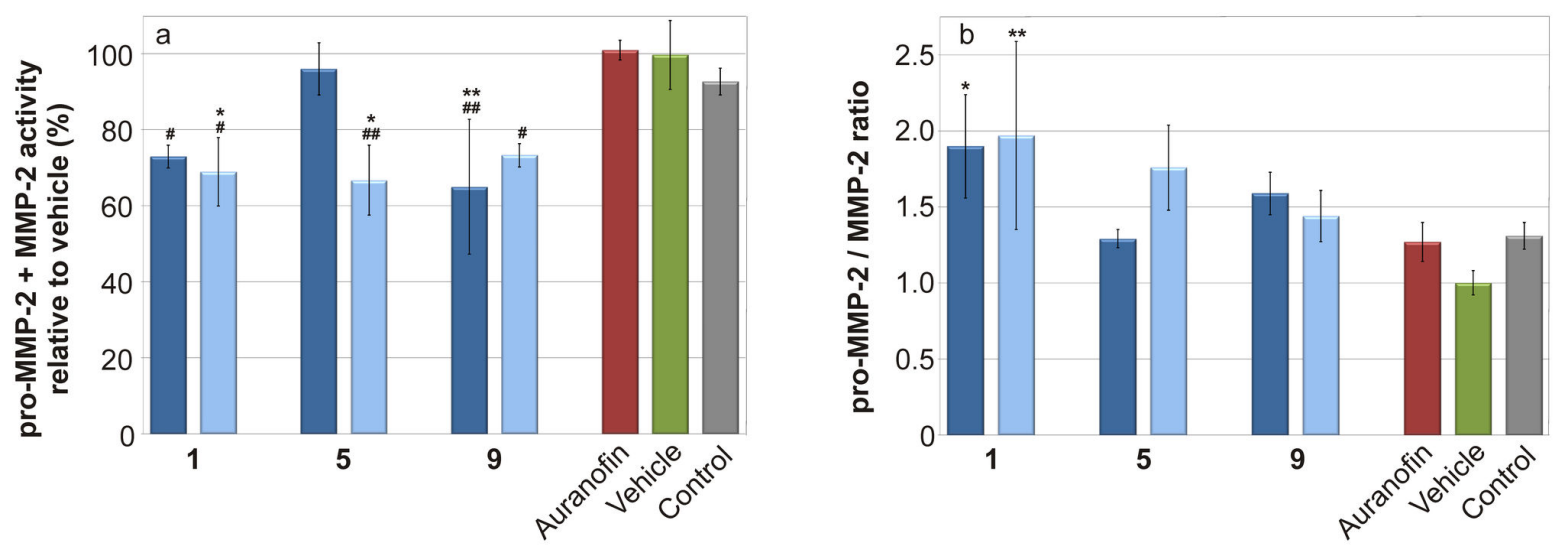
Effects of tested gold(I) complexes and the reference drug Auranofin on LPS-induced matrix metalloproteinases (MMP) activity. Cells were pretreated with the complexes 1, 5 and 9 (100 nM - depicted in dark blue; 600 nM - depicted in light blue), Auranofin (100 nM), or the *vehicle* (DMSO) only. After 1 h of the incubation, the inflammatory response was induced by LPS (except for the *control* cells). Activity of (pro-)MMP-2 was detected by zymography, (a). Intensity of digested bands was analysed by densitometry analysis. The figure shows pro-MMP-2 / MMP-2 ratio, (b). Results are expressed as means for three independent experiments. * Significant difference in comparison to vehicle-treated cells (*p* < 0.05), ** significant difference in comparison to vehicle-treated cells (*p* < 0.01), # significant difference in comparison to Auranofin-treated cells (*p* < 0.05), ## significant difference in comparison to Auranofin-treated cells (*p* < 0.01).

The other hallmark of the inflammation is the production of matrix metalloproteinases (MMPs). The activity of MMP2 and pro-MMP2 is summarised in Figure 5a. The tested gold(I) complexes were able to significantly decrease the activity of pro-MMP2 by 15–40% and to change the pro-MMP2/MMP2 ratio in the favour of physiologically inactive pro-MMP2 (Figure 5b). The activity of MMP9 was not affected by the tested compounds (data not shown). The ability of the gold(I) complexes to attenuate activity of MMP2 sketches in their anti-inflammatory potential.

### *In Vivo* Pharmacological and *Ex Vivo* Histological Evaluations of Antiedematous Activity of 1, 5 and 9

**Figure 6:**
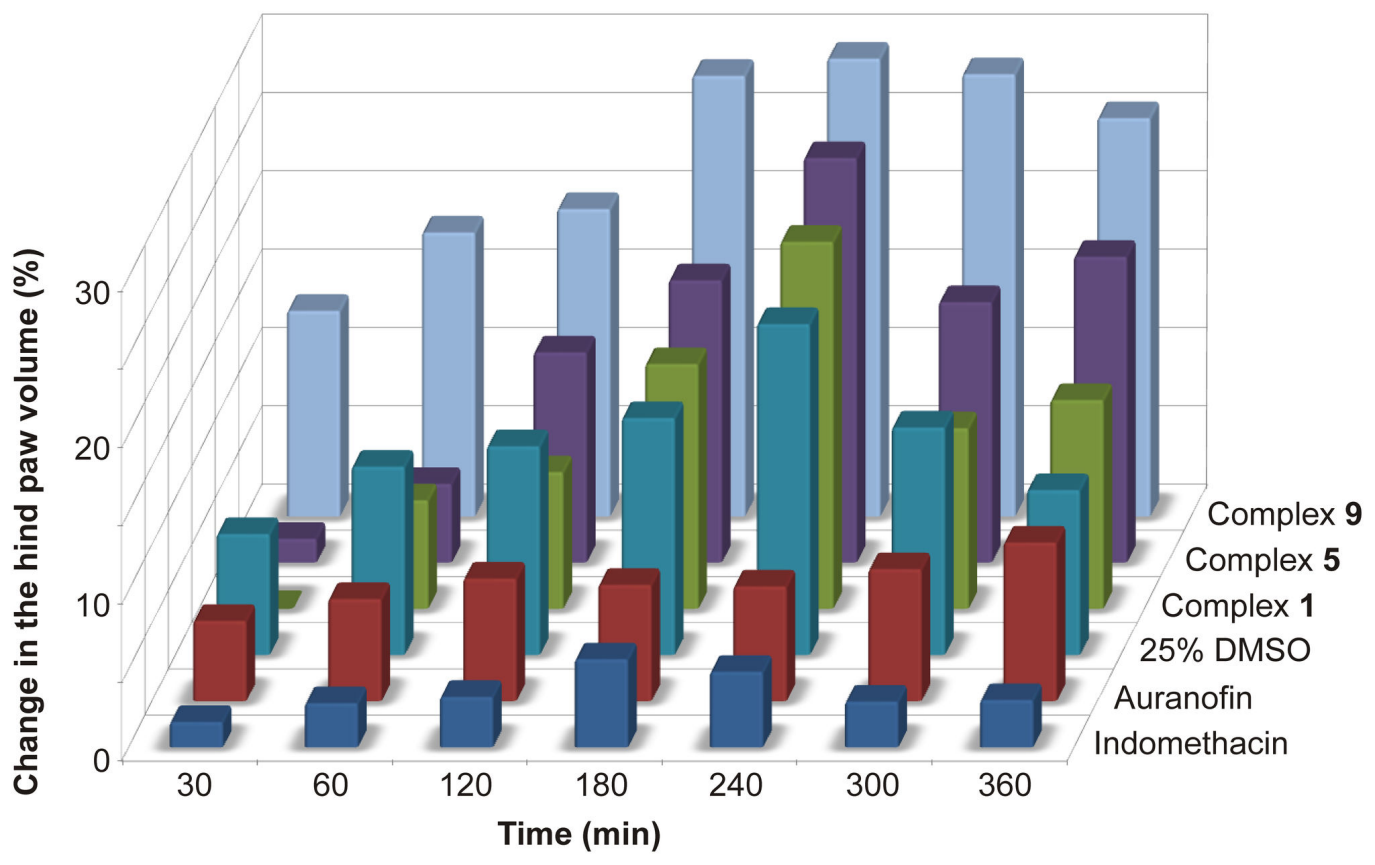
The time-dependent profile of antiedematous effect of tested compounds on carrageenan induced hind paw edema in rats.

Based on the *in vitro* experiments, the *in vivo* tests of anti-inflammatory activity of the complexes **1**, **5** and **9** were performed using the carrageenan-induced hind paw edema model, which investigate the effect of the tested complexes on one of the main symptoms of acute inflammation, i.e. the formation of edema. The clinically used gold-metallodrug Auranofin was used as the primary standard of the anti-inflammatory activity. In the experiments, we used the same dosages of Auranofin as the tested compounds; i.e. 10 mg/kg in the form of the fine suspension in 25% DMSO (v/v in water for injections PhEur) applied intraperitonealy 30 min before the intraplantar injection of carrageenan. As a secondary standard, acting dominantly by the different biochemical pathway (i.e. cyclooxygenase inhibition), the NSAID indomethacin was used in the dose of 5 mg/kg. The comprehensive overview of antiedematous activity profiles of the tested compounds is summarized in Figure 6.

In contrast to the results of *in vitro* anti-inflammatory activity, all the tested complexes showed no anti-inflammatory activity *in vivo*. The complexes **1** and **5** did not influence the swelling induced by the carrageenan injection. On the other hand, the complex **9** augmented the hind paw edema significantly, as compared with the control (25% DMSO) by the one-way ANOVA with *post-hoc* Tukey test on the level of significance *p* = 0.01. 

**Figure 7:**
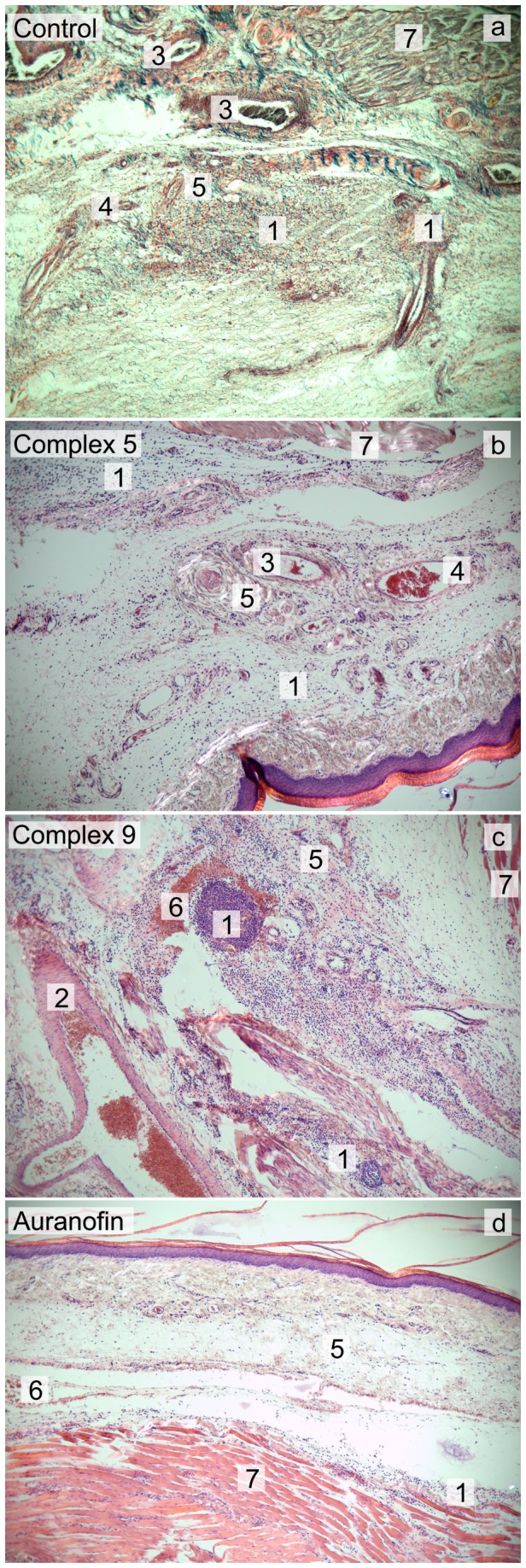
Histologic sections of the hind paw, stained with Gömöri trichrome staining (a) and Hematoxylin-eosin (b-d) (photographed at 40x magnification). Tissue exposed to 25% DMSO (control; a), and complex 5 (b) with the massive acute inflammatory reaction in the transition from dermis to hypodermis with the massive infiltrate of neurophils (PMN) in connective tissues; tissue exposed to complex 9 (c) with the massive acute inflammatory reaction reaching from the hypodermis up to the papillary layer of dermis with a massive infiltrate of neurophils (PMN); tissue exposed to Auranofin (d) with the inflammatory reaction in the hypodermis with a slight PMN infiltrate. 1 - PMN infiltrate, 2 - arteriola, 3 - dilated veins with haemostasis, 4 - vein with PMN presence in the blood stream and haemostasis, 5 - collagen connective tissue, 6 - haemorrhage characterised by the presence of erythrocytes in interstitium, 7 - muscle fibres.

To assess the tissue consequences connected with the formation of inflammation and edema and the level of influence caused by the tested compounds after the intraplantar injection of carrageenan, the histopathological observations were made on the tissue sections. The histopathological changes in tissues, stained by the standard H&E-staining, were quite similar from the qualitative point of view in all samples. The affected tissues (dermis up to papillary layer and hypodermis) clearly show the signs of inflammation, e.g. the presence of inflammation infiltrate, vasodilatation with the haemostasis, presence of leukocytes in blood stream, and extracellular edema. The noticeable differences were found in the extent of infiltration of subcutaneous tissues and dermis by the polymorphonuclear cells (PMNs, mostly neutrophils) in the samples obtained from the Auranofin and Indomethacin treated groups (see Figure 7), where only focal mobilization of PMNs in dermis is evident and the number of PMNs decreases dramatically with the proximity of epidermis. On the other hand in all other samples, obtained from the groups of animals treated by the title complexes or the control group (only vehicle, 25% DMSO, was applied), the massive infiltration of PMNs in hypodermis and dermis was observed, accompanied by the presence of PMNs up to the papillary layer (for the representative examples see Figure 7).

In addition to the classical histological investigation, the immunohistochemical detection of apoptosis (caspase 3, TUNEL assay), TNF-α, interleukin 6 (IL-6) and selectin E (CD62E) was performed. Unfortunately, the results of the immunohistochemical detection were disputable and inconclusive due to the tissue nature, because no differences were observed between the samples, standards and control, and therefore they are not discussed in detail. However, it is evident that the results of histological evaluation of tissue samples tightly correlate with the results obtained by plethysmometric measurements.

The obtained results are rather surprising due to the fact that previously reported isostructural complexes, involving the ligands not bearing the chloro-substitution at the position C2 of the purine skeleton, showed remarkable antiedematous activity, comparable with metallodrug Auranofin as well as the highly active derivatives of gold(I) complexes involving 3-(aryl)-2-sulfanylpropenoic acid [48] and higher activity than other gold(I) and gold(III) complexes [49,50]. The cause of this radical change in biological activity cannot be explained by trivial means, while the biological activity of these complexes itself is based on a multimodal effect. The main aspect influencing the quality of the action is undoubtedly the pharmacophore of Au(I)-trisubstituted phosphine derivative, however the quantity of the action is dependent from a variety of physical and chemical properties, like the stability in biological systems, influence of ligands on biodistribution and excretion of complexes, the intrinsic pro-inflammatory effect, etc.

With the intention to better understand the mechanism of interaction of the tested complexes with biological systems, we decided to perform the solution experiments involving two main sulphur-containing constituents of human plasma, amino acid cysteine and reduced glutathione. Thus, the ESI mass spectrometry was used to analyse the specific species participating in the interactions with biological systems.

### Interactions with a Mixture of Cysteine and Glutathione Assessed by ESI-MS

Due to chemical properties of Au(I) species they tend to form the strong coordination bonds with soft Lewis base ligands, such as thiolato or selenolato ions, or phosphine derivatives, while the latter ones form the most stable bonds. Therefore, Au(I) complexes tend to bind to sulfhydryl-containing substances in the biologically relevant environments (such as blood or serum), such as amino acid cysteine (Cys) or small proteins (such as glutathione - GSH) and in particular with high molecular proteins (such as albumin or globulins [51]), with the ligand exchange mechanism. The exchange of N-ligands for S-ligands occurs relatively quickly (within 20 minutes when interacting with albumin and globulins in the blood [52]), the P-ligand exchange occurs much more slowly, and it seems that in this mechanism the cooperative effects of adjacent thiolato or selenolato ligands in the neighborhood of interaction site play an important role, which is interpreted as one of the molecular mechanisms of incorporation of gold into the active site of selenium-containing flavoreductases, such as thioredoxin reductase [53].

**Figure 8:**
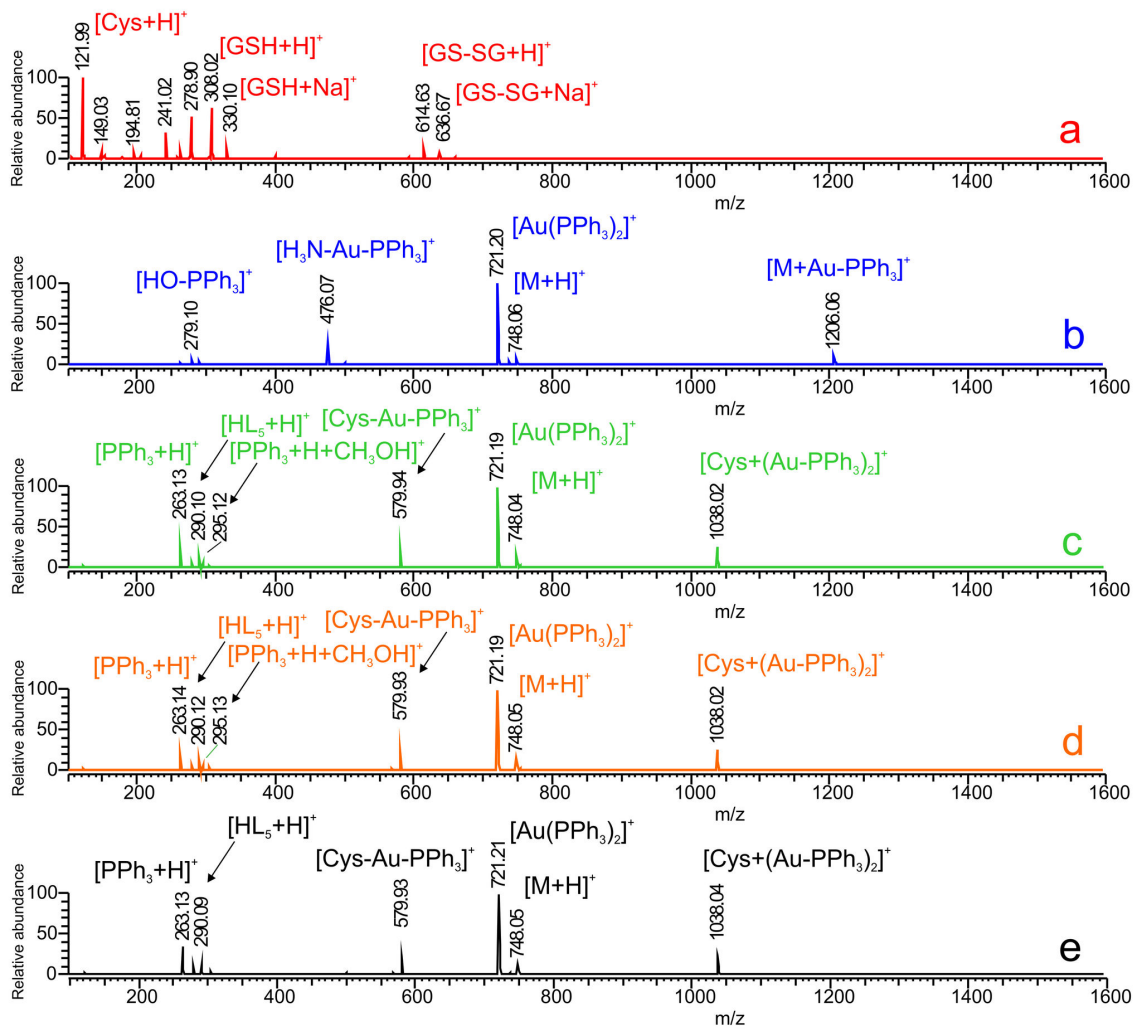
The ESI mass spectrometry analysis of interactions between the complex 5 and sulphur-containing biomolecules. The mass spectrum of the mixture of physiological concentrations of cysteine (290 μM) and reduced glutathione (6 μM), (a); the mass spectrum of the solution of complex 5, (b); the mass spectrum of the interacting system containing the physiological concentrations of cysteine and reduced glutathione and 5, immediately after mixing, (c); the mass spectrum of the interacting system containing the physiological concentrations of cysteine and reduced glutathione and 5, 1 h after mixing, (d); the mass spectrum of the interacting system containing the physiological concentrations of cysteine and reduced glutathione and 5, 12 h after mixing, (e).

In the scope of our work, we have verified the expected behaviour of selected complex **5** (applied in the concentration of 15 µM, corresponding to the highest therapeutic blood levels of gold during chrysotherapy [54]) in biologically relevant conditions [55] using a mixture of cysteine (at 290 µM concentration) and reduced glutathione (at the 6 µM concentration). Based on the results of ESI-MS experiments, we confirmed that the complex **5** reacts with sulfhydryl-containing substances in time-independent manner by the ligand-exchange mechanism based on the substitution of *N*-ligand (*N*6-benzyladenine derivative) by the cysteine molecule. This mechanism was confirmed by the emergence of ion 579.93 *m/z*, corresponding to the [Cys-Au-PPh_3_]^+^ intermediate, and ion 1038.03 *m/z*, corresponding to the ionic species [Cys-(Au-PPh_3_)_2_]^+^ (see Figure 8). In biologically relevant concentrations of both low molecular sulfhydryl-containing compounds, we observed no intermediates involving the glutathione molecule.

The interaction studies revealed also another important fact about the studied complexes. In contrast to the previously published structurally similar complexes, involving the ligands not bearing the chloro-substitution at the position C2 of the purine skeleton [10], the mass spectra of the complexes revealed a considerable instability of the complexes, as demonstrated by the appearance of the intensive ion 721.19 *m/z*, corresponding to the [Au(PPh_3_)_2_]^+^ intermediate in all solutions (in the solution of complex **5** as well as in the mixtures with cysteine and glutathione), and other ionic species involving the residue Au-PPh_3_ (e.g. [M+Au-PPh_3_]^+^; 1206.06 *m/z*), the free ligand ([HL_5_+H]^+^; 290.10 *m/z*), or the free triphenylphosphine residue (e.g. [PPh_3_+H]^+^; 263.13 *m*/*z* or [PPh_3_+H+CH_3_OH]^+^; 295.13 *m/z*). The described instability in solutions might contribute significantly to the behaviour of title complexes in biological systems leading to the inactivity in context of antiedematous activity as presented above.

## Conclusions

The gold(I) complexes of the general formula [Au(L_n_)(PPh_3_)] (1–9) involving deprotonated 2-chloro-*N*6-(substituted-benzyl)adenine derivatives (L_n_) coordinated through the N9 atom, and PPh_3_ molecule coordinated to the central atom through its phosphorus atom were prepared and characterized. The results obtained by the *in vitro* model of the LPS-activated THP-1 monocytes showed the ability of the representative complexes **1**, **5** and **9** to interfere with the cell-cycle of THP-1 cells leading to hormetic effect and simultaneously decrease the secretion of pro-inflammatory cytokines TNF-α and IL-1β as well or even better as gold(I)-based metallodrug Auranofin at the same concentration level. The prepared gold(I) complexes also showed the ability to decrease the activity of matrix metalloproteinase 2, while leaving the MMP9 unaffected. In conjunction with the promising results of *in vitro* assays, the antiedematous effect was evaluated by carrageenan-induced rat hind-paw edema model. In addition to the pharmacological observations, the affected hind paws were *post mortem* subjected to histological and immunohistochemical evaluations. The results of both *in vivo* and *ex vivo* methods, however, revealed low antiedematous and anti-inflammatory effects of the gold(I) complexes. This discrepancy may be caused by low stability of the complexes in physiological conditions, as demonstrated by the interaction studies with sulfur-containing biomolecules (cysteine and GSH) using the electrospray-ionization mass spectrometry. 

## Supporting Information

Supporting Information S1 **Figure S1.** TG/DTA curves of the complexes 1 (*left*) and 6 (*right*) given together with the calculated and observed weight losses. **Figure S2.** ^31^P NMR spectrum of complex 6.
(DOCX)
