# Supplementary material for: Effect of 2-Chloro-Substitution of Adenine Moiety in Mixed-Ligand Gold(I) Triphenylphosphine Complexes on Anti-Inflammatory Activity: The Discrepancy between the In Vivo and In Vitro Models
Source: PLoS One. 2013 Nov 27;8(11):e82441. doi: 10.1371/journal.pone.0082441 (PMC3842384; doi:10.1371/journal.pone.0082441)
Supplement: Supporting Information S1 — Figure S1. TG/DTA curves of the complexes 1 (left) and 6 (right) given together with the calculated and observed weight losses. Figure S2. 31P NMR spectrum of complex 6. (DOCX) [file pone.0082441.s001.docx]

# Effect of 2-chloro-substitution of adenine moiety in mixed-ligand gold(I) triphenylphosphine complexes on anti-inflammatory activity: The discrepancy between the *in vivo* and *in vitro* models.

Jan Hošek^1^, Ján Vančo^1^, Pavel Štarha^1^, Lenka Paráková^2^, Zdeněk Trávníček^1^*

**^1^** Department of Inorganic Chemistry, Regional Centre of Advanced Technologies and Materials, Faculty of Science, Palacký University, Olomouc, Czech Republic, **^2^**Department of Human Pharmacology and Toxicology, Faculty of Pharmacy, University of Veterinary and Pharmaceutical Sciences Brno, Brno, Czech Republic

* Corresponding author: Department of Inorganic Chemistry, Regional Centre of Advanced Technologies and Materials, Faculty of Science, Palacký University, 17. listopadu 12, CZ-771 46 Olomouc, Czech Republic. Phone: +420-585-634-352. Fax: +420-585-634-954. E-mail: zdenek.travnicek@upol.cz.

Email addresses: JH (jan.hosek@upol.cz), JV (jan.vanco@upol.cz), PŠ (pavel.starha@upol.cz), LP (parakoval@vfu.cz), ZT (zdenek.travnicek@upol.cz).

# The results of elemental analysis, ESI mass spectrometry, ^1^H and ^13^C NMR, FT-IR and Raman spectroscopies and molar conductivity measurements.

[Au(L_1_)(PPh_3_)] (**1**): Yield: 68%, Anal. calc. for C_30_H_25_N_5_ClPAu: C, 50.11%; H, 3.50%; N, 9.74%; Cl, 4.93%. Found: C, 50.35%; H, 3.64%; N, 9.83%; Cl, 5.10%. ESI+ MS (methanol, m/z) 260 (calc. 259) [HL_1_+H]^+^, 718 (717) [Au(HL_1_)(PPh_3_)]^+^. ^1^H NMR, DMF-*d_7_*, δ, ppm: 8.10, 1H, br, HN^6^, 7.99, 1H, s, HC^8^, 7.75–7.60, 15H, m, HC^PPh3^, 7.45, 2H, d, 7.5, HC^11,15^, 7.32, 2H, t, 7.5, HC^12,14^, 7.24, 1H, t, 7.5, HC^13^, 4.88, 2H, br, HC^9^. ^13^C NMR, DMF-*d_7_*, δ, ppm: 155.14 (C6), 151.94 (C2), 149.41 (C4), 146.91 (C8), 140.73 (C10), 134.61–129.88 (C^PPh3^), 128.45 (C11,15), 127.66 (C12,14), 126.90 (C13), 118.75 (C5), 43.61 (C9). ^31^P NMR, DMF-*d_7_*, δ, ppm: 32.44. IR (*ν*_ATR_/cm^–1^): 3407 ν(N–H); 3051, 2935 ν(C–H); 1606 ν(C=N)_ar_; 1001 ν(C–Cl)_ar_; 541 ν(Au–N), 365 ν(Au–P). Raman (cm^-1^): 3048, 3025, 2998 ν(C–H); 1612 ν(C=N)_ar_; 1000 ν(C–Cl)_ar_; 541 ν(Au–N); 363 ν(Au–P). *Λ*_M_ (DMF/methanol, S cm^2^ mol^-1^): 6.7/18.3.

[Au(L_2_)(PPh_3_)] (**2**): Yield: 72%, *Anal.* calc. for C_30_H_24_N_5_ClFPAu: C, 48.89%; H, 3.28%; N, 9.50%; Cl, 4.81%. Found: C, 49.35%; H, 3.56%; N, 9.74%; Cl, 5.10%. ESI+ MS (methanol, *m/z*) 278 (277) [HL_3_+H]^+^, 736 (735) [Au(HL_3_)(PPh_3_)]^+^, 758 (758) [Au(L_3_)(PPh_3_)+Na]^+^. ^1^H NMR, DMF-*d_7_*, δ, ppm: 8.31, 1H, br, HN^6^, 8.00, 1H, s, HC^8^, 7.75–7.68, 15H, m, HC^PPh3^, 7.38, 1H, qq, 7.8, 1.8, HC^15^, 7.29, 1H, d, 8.1, HC^13^, 7.27, 1H, tt, 9.6, 1.8, HC^14^, 7.05, 1H, tt, 8.4, 2.7, HC^11^, 4.91, 2H, br, HC^9^. ^13^C NMR, DMF-*d_7_*, δ, ppm: 161.75 (C12), 155.11 (C6), 151.81 (C2), 149.59 (C4), 147.87 (C8), 143.88 (C10), 134.56–128.85 (C^PPh3^), 130.39, 130.30 (C14), 123.59, 123.57 (C15), 118.75 (C5), 114.37, 114.15 (C13), 113.68, 113.47 (C11), 43.67 (C9). ^31^P NMR, DMF-*d_7_*, δ, ppm: signal not detected even after 14 hrs of the experiment due to limited solubility. IR (*ν*_ATR_/cm^–1^): 3245 ν(N–H); 3056, 3028 ν(C–H); 1611 ν(C=N)_ar_; 1264 ν(C–F)_ar_; 1102 ν(C–Cl)_ar_; 545 ν(Au–N); 359 ν(Au–P). Raman (cm^-1^): 3057, 2975, 2919 ν(C–H); 1604 ν(C=N)_ar_; 1259 ν(C–F)_ar_; 1001 ν(C–Cl)_ar_; 545 ν(Au–N); 345 ν(Au–P). *Λ*_M_ (DMF/methanol, S cm^2^ mol^-1^): 7.3/19.9.

[Au(L_3_)(PPh_3_)] (**3**): Yield: 71%, *Anal.* calc. for C_30_H_23_N_5_Cl_2_PAu: C, 47.89%; H, 3.08%; N, 9.30%; Cl, 9.42%. Found: C, 47.54%; H, 3.30%; N, 9.52%; Cl, 9.14%. ESI+ MS (methanol, *m/z*) 295 (294) [HL_4_+H]^+^, 753 (752) [Au(HL_4_)(PPh_3_)]^+^. ^1^H NMR, DMF-*d_7_*, δ, ppm: 8.07, 1H, br, HN^6^, 7.97, 1H, s, HC^8^, 7.76–7.66, 15H, m, HC^PPh3^, 7.47, 2H, m, HC^12,15^, 7.30, 2H, m, HC^13,14^, 4.92, 2H, br, HC^9^. ^13^C NMR, DMF-*d_7_*, δ, ppm: 132.51 (C11), 155.39 (C6), 151.70 (C2), 149.64 (C4), 149.10 (C8), 137.57 (C10), 134.55–129.35 (C^PPh3^), 129.39 (C15), 129.02 (C12), 128.89 (C13), 127.28 (C14), 119.35 (C5), 41.92 (C9). ^31^P NMR, DMF-*d_7_*, δ, ppm: 31.41. IR (*ν*_ATR_/cm^–1^): 3240 ν(N–H); 3055, 2939 ν(C–H); 1608 ν(C=N)_ar_; 1195 ν(C–Cl)_ar_; 1101 ν(C–Cl)_ar_; 542 ν(Au–N); 364 ν(Au–P). Raman (cm^-1^): 3059, 2991, 2936 ν(C–H); 1612 ν(C=N)_ar_; 1184 ν(C–Cl)_ar_; 1000 ν(C–Cl)_ar_; 538 ν(Au–N); 358 ν(Au–P). *Λ*_M_ (DMF/methanol, S cm^2^ mol^-1^): 4.1/15.3.

[Au(L_4_)(PPh_3_)] (**4**): Yield: 73%, *Anal.* calc. for C_30_H_24_N_5_Cl_2_PAu: C, 47.82%; H, 3.21%; N, 9.29%; Cl, 9.41%. Found: C, 47.46%; H, 3.67%; N, 9.12%; Cl, 8.93%. ESI+ MS (methanol, *m/z*) 295 (294) [HL_5_+H]^+^, 753 (752) [Au(HL_5_)(PPh_3_)]^+^. ^1^H NMR, DMF-*d_7_*, δ, ppm: 8.20, 1H, br, HN^6^, 7.96, 1H, s, HC^8^, 7.76–7.65, 15H, m, HC^PPh3^, 7.51, 1H, t, 1.9, HC^11^, 7.42, 1H, d, 7.6, HC^15^, 7.37, 1H, t, 7.4, HC^14^, 7.30, 1H, dd, 7.8, 1.9, HC^13^, 4.85, 2H, br, HC^9^. ^13^C NMR, DMF-*d_7_*, δ, ppm: 155.34 (C6), 151.74 (C2), 149.57 (C4), 149.01 (C8), 143.46 (C10), 134.57–128.74 (C^PPh3^), 133.60 (C12), 130.28 (C14), 127.54 (C11), 126.93 (C13), 126.29 (C15), 119.32 (C5), 43.48 (C9). ^31^P NMR, DMF-*d_7_*, δ, ppm: 31.37. IR (*ν*_ATR_/cm^–1^): 3239 ν(N–H); 3060, 2991, 2941 ν(C–H); 1606 ν(C=N)_ar_; 1198 ν(C–Cl)_ar_; 1001 ν(C–Cl)_ar_; 539 ν(Au–N); 360 ν(Au–P). Raman (cm^-1^): 3060, 2991, 2941 ν(C–H); 1610 ν(C=N)_ar_; 1166 ν(C–Cl)_ar_; 1000 ν(C–Cl)_ar_; 549 ν(Au–N); 357 ν(Au–P). *Λ*_M_ (DMF/methanol, S cm^2^ mol^-1^): 3.6/8.7.

[Au(L_5_)(PPh_3_)] (**5**). Yield: 72%, *Anal.* calc. for C_31_H_27_N_5_ClOPAu: C, 49.71%; H, 3.63%; N, 9.35%; Cl, 4.73%. Found: C, 50.01%; H, 3.82%; N, 9.50%; Cl, 4.29%. ESI+ MS (methanol, *m/z*) 290 (289) [HL_6_+H]^+^, 714 (713), 748 (747) [Au(HL_6_)(PPh_3_)]^+^, 771 (771) [Au(L_6_)(PPh_3_)+Na]^+^. ^1^H NMR, DMF-*d_7_*, δ, ppm: 8.02, 1H, br, HN^6^, 7.97, 1H, s, HC^8^, 7.75–7.61, 15H, m, HC^PPh3^, 7.31, 1H, d, 7.1, HC^15^, 7.24, 1H, t, 7.1, HC^13^, 7.02, 1H, d, 8.1, HC^12^, 6.88, 1H, t, 7.5, HC^14^, 4.81, 2H, br, HC^9^, 3.90, 3H, s, HC^16^. ^13^C NMR, DMF-*d_7_*, δ, ppm: 157.40 (C11), 155.75 (C6), 152.00 (C2), 148.70 (C8), 145.56 (C4), 134.61–128.61 (C^PPh3^), 127.61 (C10), 128.13 (C13), 127.59 (C15), 120.44 (C14), 119.40 (C5), 110.50 (C12), 55.30 (C16), 39.13 (C9). ^31^P NMR, DMF-*d_7_*, δ, ppm: 31.85. IR (*ν*_ATR_/cm^–1^): 3245 ν(N–H); 3056, 3028, 2954 ν(C–H); 1611 ν(C=N)_ar_; 1002m ν(C–Cl)_ar_; 544 ν(Au–N); 342 ν(Au–P). Raman (cm^-1^): 3057, 2940 ν(C–H); 1612 ν(C=N)_ar_; 1003 ν(C–Cl)_ar_; 542 ν(Au–N); 365 ν(Au–P). *Λ*_M_ (DMF/methanol, S cm^2^ mol^-1^): 1.9/19.6.

[Au(L_6_)(PPh_3_)]∙H_2_O (**6**): Yield: 69%, *Anal.* calc. for C_31_H_29_N_5_ClO_2_PAu: C, 48.54%; H, 3.81%; N, 9.13%; Cl, 4.62%. Found: C, 48.55%; H, 3.59%; N, 8.73%; Cl, 4.12%. ESI+ MS (methanol, *m/z*) 290 (289) [HL_7_+H]^+^, 714 (713) [Au(HL_7_)(PPh_3_)]^+^, 748 (747) [Au(L_7_)(PPh_3_)+Na]^+^. ^1^H NMR, DMF-*d_7_*, δ, ppm: 8.02, 1H, br, HN^6^, 7.95, 1H, s, HC^8^, 7.75–7.60, 15H, m, HC^PPh3^, 7.24, 1H, t, 7.8, HC^14^, 7.07, 1H, t, 2.2, HC^11^, 7.01, 1H, d, 7.7, HC^15^, 6.82, 1H, dd, 8.2, 2.4, HC^13^, 4.85, 2H, br, HC^9^, 3.78, 3H, s, HC^16^. ^13^C NMR, DMF-*d_7_*, δ, ppm: 160.01 (C12), 155.36 (C6), 151.67 (C2), 148.43 (C4), 148.31 (C8), 142.42 (C10), 134.57–128.94 (C^PPh3^), 129.46 (C14), 119.79 (C15), 119.20 (C5), 113.49 (C13), 112.18 (C11), 54.92 (C16), 44.00 (C9). ^31^P NMR, DMF-*d_7_*, δ, ppm: 30.97. IR (*ν*_ATR_/cm^–1^): 3233 ν(N–H); 3050, 2983, 2956, 2926 ν(C–H); 1613 ν(C=N)_ar_; 1030 ν(C–Cl)_ar_; 544 ν(Au–N); 344 ν(Au–P). Raman (cm^-1^): 3057, 3017, 2991, 2927 ν(C–H); 1611 ν(C=N)_ar_; 1000 ν(C–Cl)_ar_; 531 ν(Au–N); 354 ν(Au–P). *Λ*_M_ (DMF/methanol, S cm^2^ mol^-1^): 7.3/6.8.

[Au(L_7_)(PPh_3_)]∙H_2_O (**7**): Yield: 75%, *Anal.* calc. for C_31_H_29_N_5_ClO_2_PAu: C, 48.54%; H, 3.81%; N, 9.13%; Cl, 4.62%. Found: C, 48.16%; H, 3.61%; N, 8.85%; Cl, 4.32%. ESI+ MS (methanol, *m/z*) 290 (289) [HL_8_+H]^+^, 748 (747) [Au(HL_8_)(PPh_3_)]^+^, 771 (771) [Au(L_8_)(PPh_3_)+Na]^+^. ^1^H NMR, DMF-*d_7_*, δ, ppm: 8.02, 1H, br, HN^6^, 7.95, 1H, s, HC^8^, 7.76–7.63, 15H, m, 7.33, 2H, d, 7.8, HC^11,15^, 7.13, 2H, d, 8.0, HC^12,14^, 4.82, 2H, br, HC^9^, 2.28, 3H, s, HC^16^. ^13^C NMR, DMF-*d_7_*, δ, ppm: 155.37 (C6), 151.82 (C2), 149.38 (C4), 148.29 (C8), 137.69 (C10), 136.25 (C13), 134.59–128.86 (C^PPh3^), 129.05 (C12,14), 127.65 (C11,15), 119.12 (C5), 43.74 (C9), 20.61 (C16). ^31^P NMR, DMF-*d_7_*, δ, ppm: signal not detected even after 14 hrs of the experiment due to limited solubility. IR (*ν*_ATR_/cm^–1^): 3228 ν(C–H); 3049, 2988, 2919 ν(C–H); 1608 ν(C=N)_ar_; 1110 ν(C–Cl)_ar_; 543 ν(Au–N); 362 ν(Au–P). Raman (cm^-1^): 3056, 2921 ν(C–H); 1613 ν(C=N)_ar_; 1000 ν(C–Cl)_ar_; 536 ν(Au–N); 371 ν(Au–P). *Λ*_M_ (DMF/methanol, S cm^2^ mol^-1^): 4.1/24.7.

[Au(L_8_)(PPh_3_)] (**8**): Yield: 70%, *Anal.* calc. for C_30_H_25_N_5_ClOPAu: C, 49.02%; H, 3.42%; N, 9.52%; Cl, 4.82%. Found: C, 49.28%; H, 3.24%; N, 9.85%; Cl, 4.96%. ESI+ MS (methanol, *m/z*) 276 (275) [HL_9_+H]^+^, 734 (733) [Au(HL_9_)(PPh_3_)]^+^. ^1^H NMR, DMF-*d_7_*, δ, ppm: 9.59, 1H, br, HO^16^, 8.02, 1H, br, HN^6^, 7.91, 1H, s, HC^8^, 7.74–7.65, 15H, m, HC^PPh3^, 7.26, 2H, d, 8.4, HC^11,15^, 6.80, 2H, d, 8.2, HC^12,14^, 4.76, 2H, br, HC^9^. ^13^C NMR, DMF-*d_7_*, δ, ppm: 157.25 (C13), 155.30 (C6), 151.59 (C2), 150.51 (C4), 148.93 (C8), 134.54–128.86 (C^PPh3^), 129.03 (C11,15), 119.30 (C5), 115.28 (C12,14), 43.70 (C9). ^31^P NMR, DMF-*d_7_*, δ, ppm: 31.38. IR (*ν*_ATR_/cm^–1^): 3319 ν(N–H); 3052 ν(C–H); 1606 ν(C=N)_ar_; 1101 ν(C–Cl)_ar_; 541 ν(Au–N); 359 ν(Au–P). Raman (cm^-1^): 3056, 2952 ν(C–H); 1612 ν(C=N)_ar_; 1000 ν(C–Cl)_ar_; 555 ν(Au–N); 372 ν(Au–P). *Λ*_M_ (DMF/methanol, S cm^2^ mol^-1^): 1.4/12.4.

[Au(L_9_)(PPh_3_)] (**9**): Yield: 76%, *Anal.* calc. for C_31_H_26_N_5_ClPAu: C, 50.86%; H, 3.58%; N, 9.56%; Cl, 4.84%. Found: C, 51.15%; H, 3.82%; N, 9.91%; Cl, 4.92%. ESI+ MS (methanol, *m/z*) 274 (273) [HL_10_+H]^+^, 732 (731) [Au(HL_10_)(PPh_3_)]^+^, 754 (754) [Au(L_10_)(PPh_3_)+Na]^+^. ^1^H NMR, DMF-*d_7_*, δ, ppm: 8.03, 1H, br, HN^6^, 7.94, 1H, s, HC^8^, 7.73–7.68, 15H, m, HC^PPh3^, 7.33, 2H, d, 7.8, HC^11,15^, 7.13, 2H, d, 7.8, HC^12,14^, 4.81, 2H, br, HC^9^, 2.28, 3H, s, HC^16^. ^13^C NMR, DMF-*d_7_*, δ, ppm: 155.49 (C6), 151.80 (C2), 149.37 (C4), 148.87 (C8), 137.69 (C10), 136.23 (C13), 134.56–128.79 (C^PPh3^), 129.04 (C12,14), 127.64 (C11,15), 119.27 (C5), 43.69 (C9), 20.49 (C16). ^31^P NMR, DMF-*d_7_*, δ, ppm: 31.52. IR (*ν*_ATR_/cm^–1^): 3253 ν(C–H); 3051, 2954 ν(C–H); 1608 ν(C=N)_ar_; 1003 ν(C–Cl)_ar_; 541 ν(Au–N); 361 ν(Au–P). Raman (cm^-1^): 3059, 2990, 2956 ν(C–H); 1618 ν(C=N)_ar_; 1001 ν(C–Cl)_ar_; 538 ν(Au–N); 368 ν(Au–P). *Λ*_M_ (DMF/methanol, S cm^2^ mol^-1^): 2.8/27.6.


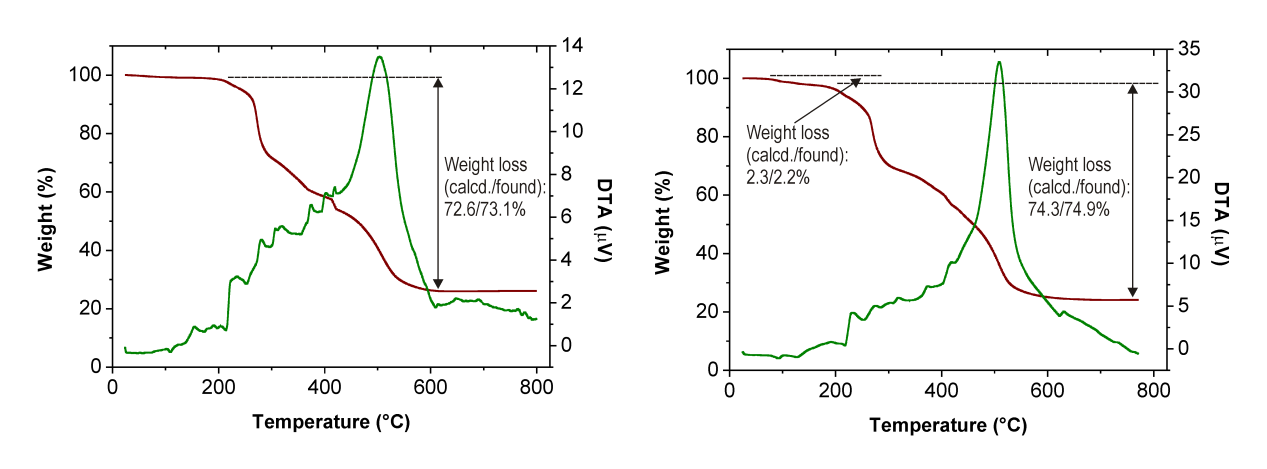


**Figure S1.** TG/DTA curves of the complexes **1** (*left*) and **6** (*right*) given together with the calculated and observed weight losses.

**
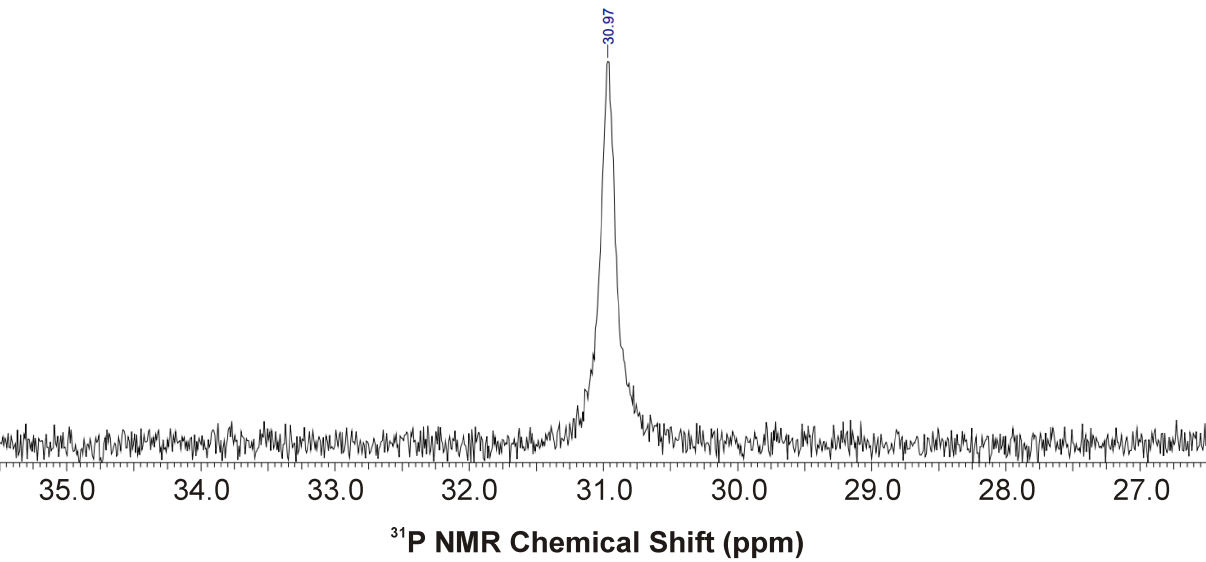
**

**Figure S2.** ^31^P NMR spectrum of complex **6**.
